# Skin and soft tissue infections in the elderly

**DOI:** 10.1097/QCO.0000000000000907

**Published:** 2023-01-25

**Authors:** Marco Falcone, Giusy Tiseo

**Affiliations:** Infectious Diseases Unit, Department of Clinical and Experimental Medicine, Azienda Ospedaliera Universitaria Pisana, University of Pisa, Pisa, Italy

**Keywords:** atypical presentation, elderly, long-acting antibiotics, methicillin-resistant Staphylococcus aureus, skin and soft tissue infections, pressure ulcer

## Abstract

To highlight the peculiarity of skin and soft tissue infections (SSTIs) in elderly patients and to provide useful elements for their optimal management.

**Recent findings:**

In the COVID-19 era, early discharge from the hospital and implementation of outpatient management is of key importance.

**Summary:**

Elderly patients are at high risk of SSTIs due to several factors, including presence of multiple comorbidities and skin factors predisposing to infections. Clinical presentation may be atypical and some signs of severity, such as fever and increase in C-reactive protein, may be absent or aspecific in this patients population. An appropriate diagnosis of SSTIs in the elderly is crucial to avoid antibiotic overtreatment. Further studies should explore factors associated with bacterial superinfections in patients with pressure ulcers or lower limb erythema. Since several risk factors for methicillin-resistant *Staphylococcus aureus* (MRSA) may coexist in elderly patients, these subjects should be carefully screened for MRSA risk factors and those with high risk of resistant etiology should receive early antibiotic therapy active against MRSA. Physicians should aim to several objectives, including clinical cure, patient safety, early discharge and return to community. SSTIs in the elderly may be managed using long-acting antibiotics, but clinical follow-up is needed.

## INTRODUCTION

The World Health Organization (WHO) predicts that the number of people aged >60 years will rise from 900 million to 2 billion between 2015 and 2050 [1]. Moreover, the Review on Antimicrobial Resistance, commissioned by the UK Government, argued that infections caused by antimicrobial resistance could kill 10 million people per year by 2050, more than the number of deaths caused by cancer [2]. Considering these two statements, it is easy to understand that in the next future elderly will represent the population at highest risk of infections and poor outcome from infectious diseases. Elderly patients have specific peculiarities that influence the presentations of infectious diseases and the response to treatments. Multiple comorbidities, changes in drug pharmacokinetics (PK) and pharmacodynamics (PD), and the presence of polypharmacy with the inherent risk of adverse drug reactions and drug–drug or drug–disease interactions make the management of infections very challenging in elderly patients [3^▪^]. Skin and soft tissue infections (SSTIs) are common in these subjects [4]. An increase of 22% of ambulatory care and a 40% of Emergency Department visits for SSTIs has been reported from 2000 to 2012 [5]. More specifically, SSTIs increased almost two-fold in older adults (>65 years of age) [5]. Moreover, the prevalence of SSTIs as cause of healthcare-associated infections ranges from less than 1% to about 20% according to different hospital settings [6], with the highest prevalence reported in acute care hospitals and long-term care facilities (10.9% and 17%, respectively) [7,8].

The clinical spectrum of skin infections is highly variable and ranges from mild infections to life-threatening diseases. The new definition of acute bacterial skin and skin structure infections (ABSSSI) has been released to allow homogeneity in patients included in clinical trials. However, some important disease entities are excluded from this definition. As a matter of fact, the guidance about ABSSSI management from Food and Drug Administration (FDA) does not address infections needing more complex treatment regimens, such as those resulting from animal or human bites, necrotizing fasciitis, diabetic foot infection, decubitus ulcer infection, myonecrosis, and ecthyma gangrenosum [9]. Some of these manifestations, such as decubitus ulcer infections are frequent in elderly patients. Moreover, elderly are usually excluded from randomized controlled trials [10]. Thus, in this category of patients using the term ABSSSI may have limitations and may be not appropriate. In this review we use the term SSTIs and discuss all type of skin infections, including those not included in the definition of ABSSSI.

**FIGURE 1:**
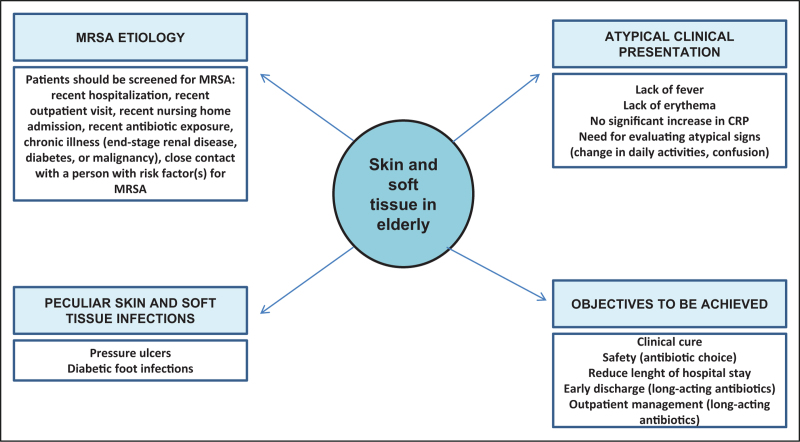
Peculiar considerations in elderly patients with skin and soft tissue infections. CRP, C-reactive protein; MRSA, methicillin-resistant *Staphylococcus aureus*.

The aim of this document is to highlight the peculiarity of SSTIs in elderly patients and to provide useful elements for their optimal management. Figure 1 summarizes peculiar elements of elderly patients with skin and soft tissue infection highlighted in this review. 

**Box 1:**
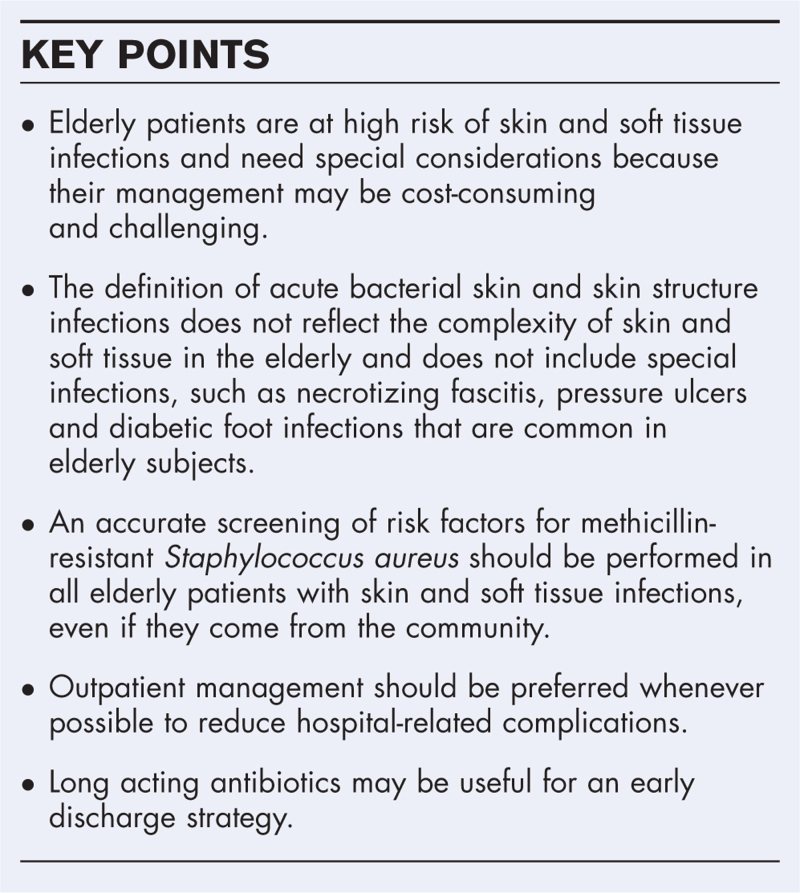
no caption available

## PECULIAR RISK FACTORS

Elderly patients have several risk factors for SSTIs. Changes in skin consistence and immunosenescence predispose to SSTIs in elderly subjects. Dermal matrix alterations, atrophy, senescence of dermal cells such as fibroblasts, and decreased synthesis and accelerated break down of dermal collagen fibers are factors that contribute to an increased susceptibility to skin infections [11]. Elderly patients have a high frequency of conditions associated with skin fragility, such as edema and trauma.

Moreover, the presence of multiple comorbidities may impact on the progression and the course of SSTIs and represents a prognostic factor in elderly patients. Among comorbidities, the presence of heart failure, one of the most common conditions in the elderly, may be associated with peripheral edema that increases the risk of erisypela and cellulitis. It has been estimated that one third of adults aged 65 or older have diabetes mellitus [12]. A retrospective study conducted in the United States from 2005 to 2010 showed that abscesses, cellulitis, and other skin infections were more frequent in patients with diabetes mellitus [13]. Moreover, patients with diabetes mellitus had a fourfold risk of skin complications compared to nondiabetic patients. Several mechanisms, such as hyperglycemia, nonenzymatic glycation of proteins, impairment of the immune response and generation of reactive oxygen species are involved in the relationship between SSTIs and diabetes mellitus [14^▪^]. Elderly patients with diabetes are more likely to develop diabetes-related complications that further complicate the management of these patients and reduce the probabilities to achieve a clinical cure from a SSTI.

Finally, the presence of malnutrition plays a crucial role in elderly patients and increases the risk of SSTIs. Malnutrition is a very common problem that affects approximately 30–50% of all hospitalized patients. Among the elderly residents in long-term care facilities, malnutrition ranges from 12.5% to 78.9% [15]. Of note, among patients who are not malnourished on admission, about one third may become malnourished while in the hospital. This evenience increases the risk of SSTIs during the hospitalization of elderly patients and may represent a challenge since elderly with malnutrition have low chances of survive and recover from an infectious disease episode.

## DIFFERENT ETIOLOGY COMPARED TO ADULTS?

Skin and soft tissue infections are usually caused by *Streptococcus pyogenes* and *Staphylococcus aureus*, including methicillin-resistant *Staphylococcus aureus* (MRSA) strains [16–19]. Less commonly identified bacteria include other *Streptococcus* species, *Enterococcus faecalis*, and Gram-negative bacteria [20,21]. MRSA has become a significant healthcare problem, especially in hospital settings. In the past decade, this organism has emerged in the community known as community-associated MRSA (CA-MRSA). Outbreaks of CA-MRSA infections have been increasingly reported worldwide with skin and soft tissue as the most common manifestation [22,23]. In the United States, the prevalence of SSTIs caused by CA-MRSA has been reported to range from 15% to 74% of all SSTIs [24]. Thus, the first consideration is to understand whether elderly patients have higher risk to have a SSTI caused by MRSA compared to adults. As a matter of fact, identification of risk factors is important in initial decisions about antibiotic selection. Risk factors for MRSA acquisition included the following factors: recent hospitalization, recent outpatient visit (within 12 months), recent nursing home admission (within 12 months), recent antibiotic exposure (range, 1–12 months), chronic illness (such as end-stage renal disease, diabetes, or malignancy), injection drug use, and close contact with a person with risk factor(s) for MRSA acquisition [25]. Living in an area with a high prevalence of CA-MRSA or admission to a hospital with a high prevalence of hospital acquired-MRSA is also considered a significant risk factor for MRSA colonization. Advancing age by itself is not considered a risk factor for MRSA infection. A study from UK studied the prevalence of MRSA in a general practice in London [26]. Among 258 older subjects living in their own home, MRSA was found in two participants (0.78%) and past history of MRSA was the only significant risk factor for MRSA colonization. However, elderly patients may have more frequently other risk factors for MRSA. Moreover, age >65 years is a significant risk factor for hospitalization and poor outcome due to a MRSA infection [27]. Hence, advancing age is indirectly linked to MRSA acquisition. Interestingly, a recent retrospective study conducted in patients >60 years old in Poland showed that the prevalence of MRSA is significantly different in categories of patients: 14.1% in young old (60–74 years), 19.5% in old old (75–85 years) and 26.7% in longevity (≥85 years old) [28].

Moreover, elderly patients residing in nursing homes appear to be at increased risk of colonization and infection by antibiotic resistant pathogens (such as MRSA). Marwick *et al.*[29] showed that the incidence of resistant bacteria is higher in nursing home patients compared to those reported in the control group (70% versus 36%, *P* = 0.026), and isolation of any resistant organism and receipt of initial inadequate antibiotic therapy are both factors independently associated with death.

These findings highlight some important considerations: first, elderly patients admitted to the hospital for SSTIs should be carefully screened for risk factors for CA-MRSA; second, the knowledge of updated epidemiological data about the CA-MRSA prevalence in elderly patients in specific areas is crucial to better decide empirical treatment for severe SSTIs; third, studies evaluating the prevalence of MRSA colonization and infections in nursing home residents are needed.

## CLINICAL PRESENTATION

The clinical presentation of SSTIs in the elderly may differ from that of adults. Systemic symptoms and signs that are typically associated with infections in younger adults, such as fever, might be absent in older people [30]. Poor vascular supply, more common in elderly, can reduce the presence of erythema, warmth and tenderness that are typically associated with skin infections. Moreover, increase in C-reactive protein may be less pronounced in this category of patients due to impaired immune system and low capacity to respond to external stressors. Unfortunately, there is a paucity of guidance to help clinicians in illuminating these nonclassical presentations [31]. A systematic review of studies assessing the diagnostic accuracy of symptoms and signs of bacterial skin infections in subjects aged over 65 years showed that the presence of pressure sores, wounds and ulcers are helpful predictors of skin infections in these patients, while other skin manifestations such as warmth and erythema were not explored in clinical studies [31]. The diagnosis of bacterial skin infections based only on the presence of wounds, pressure sores or skin ulcers may increase the risk of unnecessary antimicrobial treatment and inducing antibiotic resistance. Similarly, positive blood cultures may be misleading and should be performed in selected patients to avoid overtreatment when potential contaminants are detected [32]. Clinical presentation of skin infections in the elderly should be further explored and the occurrence of atypical signs, such as confusion and changes in activities of daily living, should be studied in this patients’ population.

Another challenge in the management of SSTIs in the elderly is to efficiently differentiate cases that are potentially lifethreatening and warrant prompt hospitalization from those that are less severe and can be managed in an outpatient setting. The Laboratory Risk Indicator for Necrotizing Fasciitis (LRINEC) scoring system has been developed to discriminate necrotizing fascitis from other severe soft-tissue infections [33]. However, its sensitivity ranges from 43.2% to 80.0% in different studies. This heterogeneity may be due to several factors, including differences in race, region or area, demographics (age, sex, body mass index), comorbid medical conditions (diabetes, immunosuppressant status) [34]. In elderly patients, some items of the LRINEC score may be not present (increase in C-reactive protein or white blood cell) while other items may be altered due to other conditions (hyponatremia, increase in creatinine and glucose values). Further studies are needed to validate existing scores or develop new dedicated scores for elderly with severe skin infections.

## DON’T FORGET SPECIAL SKIN AND SOFT TISSUE INFECTIONS

Pressure ulcers are common in elderly patients, especially in those who live in long-term facilities. In community settings there is a lower rate of pressure ulcers occurrence due to less immobility and malnutrition from advanced chronic illness. The reported prevalence of pressure ulcers in the community is less than 2% of elderly individuals but progressively increase with age (4.2% in patients ≥85 years old) [35]. In the hospital setting, this data significantly increases with prevalence as high as 50% [36]. The majority of pressure ulcers occurring in hospitals develop during the initial 5 days of hospitalization and have an adverse impact on patient outcome, quality of life, length of hospital stay and healthcare costs. The management of this type of infections in the elderly is challenging. Etiology of infections of pressure ulcers differ from that of other type of skin infections. Most pressure ulcer infections are polymicrobial. Gram negative bacilli and anaerobes, such as such as *Bacteroides fragilis*, *Peptostreptococcus*, and *Clostridium* spp., are common causes of infections of sacral pressure ulcers.

Another specific type of SSTIs that may frequently occur in elderly patients is diabetic foot infections (DFIs). Diabetes mellitus is a common condition in elderly patients. The management of DFIs in elderly patients may be challenging because of specific etiology, increase in resistant organisms, need for frequent medications and challenge in antibiotic treatment. The presence of concomitant clinical conditions, such as kidney failure, and the polypharmacy should be carefully evaluated in the selection of the optimal antibiotic treatment in these patients.

## SPECIAL CONSIDERATIONS FOR TREATMENT: CURRENT CHALLENGES AND FUTURE RESEARCH

Antibiotic treatment of elderly patients with SSTIs may be challenging and the therapeutic choice may differ from that of adult patients. Special considerations should be done.

The choice of antibiotic treatment in elderly patients needs special attention considering the presence of multiple comorbidities and the high risk of toxicity. Changes in drug PK/PD and the presence of polypharmacy with the inherent risk of adverse drug reactions and drug–drug or drug–disease interactions make the choice of the optimal antibiotic very challenging in these patients [3^▪^]. Appropriate antibiotic prescription, either in terms of drug choice or dosage, is of paramount importance among elderly, but balancing efficacy, safety, tolerability and development of antimicrobial resistance is difficult [3^▪^]. Table 1 summarizes antibiotics for SSTIs and their potential limitations in elderly patients. Some oral antibiotics, including fluoroquinolones and linezolid, are associated with high risk of adverse events in the elderly: fluoroquinolones may induce cardiac adverse events and delirium in the elderly, while linezolid may cause bone marrow suppression and thrombocytopenia. Intravenous antibiotics against MRSA should be carefully selected in these patients. Vancomycin is associated with high risk of kidney failure and should be avoided in patients with chronic kidney disease.

**Table 1:** Antibiotics for skin and soft tissue infections and their use in elderly patients

Antibiotic	Coverage	Route of administration	Considerations in elderly patients
Minocycline/doxicycline	Gram-positive bacteria	Oral	No adjustment for renal function
Clindamycin	Gram-positive bacteria, anaerobes	i.v. or oral	High risk of *C. difficile* infection
Fluoroquinolones	Gram-positive bacteria, *Pseudomonas aeruginosa*	i.v. or oral	Risk of cardiac side effects (prolonged QT, arrythmias) and risk of neurological adverse event (confusion, delirium)
Linezolid	Staphylococci (incl. MRSA) Enterococci (incl. VRE), Streptococci	i.v. or oral	Risk of thrombocytopenia and myelosuppression
Glycopeptides (vancomycin/teicoplanin)	Gram-positive bacteria, Staphylococci (incl. MRSA), Enterococci (exl. VRE)	i.v.	Risk of kidney injury (vancomycin)
Daptomycin	Gram-positive bacteria	i.v.	Risk of rhabdomyolysis (consider drug-drug interactions)
Tigecycline	Active against many Gram-positive bacteria, Gram-negative bacteria (no *Pseudomonas*) and anaerobes	i.v.	Less tolerated (nausea, vomiting)
Ceftaroline	Active against Gram-positive bacteria, Enterobacteriaceae (no ESBL) No activity vs. *Pseudomonas*	i.v.	
Dalbavancin	Active against Gram-positive bacteria Staphylococci (incl. MRSA)	i.v.	Advantage: Long acting, single administration
Oritavancin	Active against Gram-positive bacteria Staphylococci (incl. MRSA), Enterococci incl VRE	i.v.	Advantage: Long acting, single administration

i.v., intravenous; MRSA, methicillin-resistant *S. aureus*; VRE, vancomycin resistant Enterococci.

Another important aspect is the objective to be achieved. Two important objectives in these patients are early discharge and early switch-therapy. A short duration of antibiotic course may be favorable due to a potential reduction of adverse events and antibiotic resistance, and the opportunity to enhance patients’ compliance and to decrease healthcare costs. Early discharge is particularly important in elderly patients to allow return to common activities. Hospitalization in elderly patients may be associated with several complications. These conditions may include worsening of preexisting issues such as mobility and cognitive impairment, but may be also be represented by new onset complication developing during hospitalization, such as delirium, hospital-acquired incontinence, falls, pressure injuries and new functional impairments [37]. Moreover during the COVID-19 pandemic, avoiding hospitalization is important to guarantee patients’ safety and reduce hospital overcrowding [38,39].

Long-acting antibiotics, including dalbavancin and oritavancin, may be useful in these patients to avoid hospital admission and its related complications. Moreover, in patients with polypharmacy the use of single administration of dalbavancin or oritavancin increases the compliance and reduces the risk of failure. A recent study described the efficacy and safety of dalbavancin among elderly patients with different type of infections, including bones and joint infections, surgical site infection, and infective endocarditis. Clinical cure was confirmed for 79% of old patients at 1, 3, and 6 months. Six adverse events (9%) were reported after dalbavancin's administration, but each time in combination with other antibiotics [40,41].

The therapeutic management of an elderly patient with SSTIs should be individualized according to the patient's profile. Patients with clinical stability, stable comorbid illness and stable social situations may be early discharged and considered for a switch to oral therapy with an antimicrobial agent with a good safety profile and simple route of administration. Conversely, in a patient, who may be defined as “complex enough,’ ’ with multiple comorbidities, polypharmacy, need for intravascular devices for drug administration, the objective should be treatment simplification [42^▪▪^]. These patients may be candidates to long-acting antibiotics also in the Emergency Department to allow early return to community. Outpatients management with the possibility to complete the follow-up of patients who received long-acting antibiotics and are early discharged should be implemented.

## CONCLUSION

Elderly patients have peculiar characteristics and the management of infectious diseases may be challenging in this patients population. The term ABSSSIs does not reflect the complexity of skin infections in elderly patients. Some entities, such as pressure ulcers and decubitus, occur frequently in these patients and need special awareness. Most elderly patients are characterized by multiple comorbidities and high risk for polypharmacy interactions. An accurate identification of patients at risk for infections caused by difficult-to-treat microorganisms such as MRSA is necessary, as well as a correct management including the early empirical administration of an active antibiotic therapy. The choice of antibiotic therapy should take in account various factors, including safety of the delivered drugs, interactions, patient profile, and possibility of permitting an early discharge, thus minimizing the costs of hospitalization. The use of long-acting antibiotics should be evaluated in these patients to avoid hospitalization and its related complications. Future research should focus on atypical presentation of SSTIs in the elderly and on the identification of patients who need antibiotic therapy and avoid antibiotic misuse. The discrimination of contamination or colonization and the presence of bacterial superinfection in patients with special SSTI such as pressure ulcers, is crucial in these cases and specific diagnostic work-up should be developed. Moreover, outpatient management should be strengthened and stakeholders and institution should be involved to implement this outpatient care.

## Acknowledgements

*None.*

### Financial support and sponsorship

*None.*

### Conflicts of interest

*Giusy Tiseo declare honoraria for educational meetings by Shionogi. Marco Falcone received unconditional grants/or speaker honoraria from MSD, Angelini, Shionogi, Pfizer, Menarini, Termo-Fisher, Gilead and Nordic Pharma. Declared conflicts of interest are outside the submitted work and did not affect the scientific objectivity of this review.*

## REFERENCES AND RECOMMENDED READING

Papers of particular interest, published within the annual period of review, have been highlighted as:

▪ of special interest
▪▪ of outstanding interest
